# Identification of Sialyltransferase 8B as a Generalized Susceptibility Gene for Psychotic and Mood Disorders on Chromosome 15q25-26

**DOI:** 10.1371/journal.pone.0038172

**Published:** 2012-05-31

**Authors:** Erica Z. McAuley, Anna Scimone, Yash Tiwari, Giti Agahi, Bryan J. Mowry, Elizabeth G. Holliday, Jennifer A. Donald, Cynthia Shannon Weickert, Phillip B. Mitchell, Peter R. Schofield, Janice M. Fullerton

**Affiliations:** 1 Psychiatric Genetics, Neuroscience Research Australia, Sydney, New South Wales, Australia; 2 School of Medical Sciences, University of New South Wales, Sydney, New South Wales, Australia; 3 Developmental Neurobiology, Schizophrenia Research Institute, Sydney, New South Wales, Australia; 4 Genetics, Queensland Centre for Mental Health Research, Brisbane, Queensland, Australia; 5 Queensland Brain Institue, University of Queensland, Brisbane, Queensland, Australia; 6 Department of Biological Sciences, Macquarie University, Sydney, New South Wales, Australia; 7 School of Psychiatry, University of New South Wales, Sydney, New South Wales, Australia; 8 Black Dog Institute, Prince of Wales Hospital, Sydney, New South Wales, Australia; University of Illinois at Chicago, United States of America

## Abstract

We previously identified a significant bipolar spectrum disorder linkage peak on 15q25-26 using 35 extended families with a broad clinical phenotype, including bipolar disorder (types I and II), recurrent unipolar depression and schizoaffective disorder. However, the specific gene(s) contributing to this signal had not been identified. By a fine mapping association study in an Australian case-control cohort (n = 385), we find that the sialyltransferase 8B (*ST8SIA2*) gene, coding for an enzyme that glycosylates proteins involved in neuronal plasticity which has previously shown association to both schizophrenia and autism, is associated with increased risk to bipolar spectrum disorder. Nominal single point association was observed with SNPs in *ST8SIA2* (rs4586379, *P* = 0.0043; rs2168351, *P* = 0.0045), and a specific risk haplotype was identified (frequency: bipolar vs controls = 0.41 vs 0.31; χ^2^ = 6.46, *P* = 0.011, OR = 1.47). Over-representation of the specific risk haplotype was also observed in an Australian schizophrenia case-control cohort (n = 256) (χ^2^ = 8.41, *P* = 0.004, OR = 1.82). Using GWAS data from the NIMH bipolar disorder (n = 2055) and NIMH schizophrenia (n = 2550) cohorts, the equivalent haplotype was significantly over-represented in bipolar disorder (χ^2^ = 5.91, *P* = 0.015, OR = 1.29), with the same direction of effect in schizophrenia, albeit non-significant (χ^2^ = 2.3, *P* = 0.129, OR = 1.09). We demonstrate marked down-regulation of ST8SIA2 gene expression across human brain development and show a significant haplotype×diagnosis effect on *ST8SIA2* mRNA levels in adult cortex (ANOVA: F(1,87) = 6.031, *P* = 0.016). These findings suggest that variation the *ST8SIA2* gene is associated with increased risk to mental illness, acting to restrict neuronal plasticity and disrupt early neuronal network formation, rendering the developing and adult brain more vulnerable to secondary genetic or environmental insults.

## Introduction

Bipolar disorder and schizophrenia are severe heritable mental illnesses. Epidemiological and genetic studies [1], [2] suggest that bipolar disorder and schizophrenia share common risk factors. Candidate schizophrenia susceptibility genes identified first through linkage, including *DISC1* [3], *DAOA* [4], and *NRG1* [5], have subsequently shown association to bipolar disorder [6], [7], [8], providing evidence that these related but clinically distinct illnesses share genetic risk. Recent GWAS studies have identified genes that contribute to disease risk for both illnesses, for example *ZNF804A* [9] and *CACNA1C* [10]; however, as these genes each contribute around 1% of the phenotypic variance, much of the shared genetic risk remains unexplained. It may be that part of the ‘missing heritability’ lies in highly penetrant, family specific risk factors [11], and that traditional linkage approaches in either schizophrenia or bipolar families may identify specific high-penetrance risk genes, which can then be interrogated in larger population-based cohorts to determine their effect at a population level.

We previously reported evidence for a bipolar disorder susceptibility locus within a 17 cM (6.2 Mb) interval on chromosome 15q25-26 through a genome-wide linkage analysis in 35 Australian multi-generational bipolar disorder families with a broad spectrum of clinical diagnoses, including major depressive disorder and schizoaffective disorder-manic type [12]. The 6.2 Mb linkage interval (86.63–92.82 Mb: NCBI Build 36.1) contains over 50 known genes, many of which are expressed in the brain. Haplotype narrowing in the 35 Australian pedigrees using an autosomal dominant model for a highly penetrant gene of large effect implicated a 2.5 Mb high priority region (90.32–92.82 Mb) [12], which contains 3 hypothetical genes and 5 protein coding genes, namely: *SLCO3A1*, *ST8SIA2*, *CHD2*, *RGMA* and *MCTP2*. Of these, the alpha-2,8-sialyltransferase 2 gene (*ST8SIA2*) – an enzyme responsible for protein glycosylation – has previously been implicated as a putative susceptibility gene for schizophrenia [13], and autism spectrum disorder (verbal subtype) [14].

We conducted a fine-mapping association study of the susceptibility locus on 15q25-26 in an Australian case-control cohort, providing evidence for association of *ST8SIA2* with bipolar disorder. The specific risk haplotype was then investigated for association in three independent case-control cohorts with either bipolar disorder or schizophrenia from Australia and the USA. Further, we explore the temporal expression profile of *ST8SIA2* over postnatal human development in the dorsolateral prefrontal cortex, and investigate the effect of the associated haplotypes on global *ST8SIA2* gene expression in the adult human dorsolateral prefrontal cortex.

## Results

### Genotyping and association analysis in the Australian bipolar disorder cohort

From a pool of 376 genotyped SNPs, 23 failed quality control measures (93.9% pass) and 4 failed the Hardy-Weinberg equilibrium test, and were omitted from the results. Eight individuals (2 BPI; 6 controls) were removed from analyses (97.9% pass) due to low genotype call rates (10% missingness).

**Figure 1:**
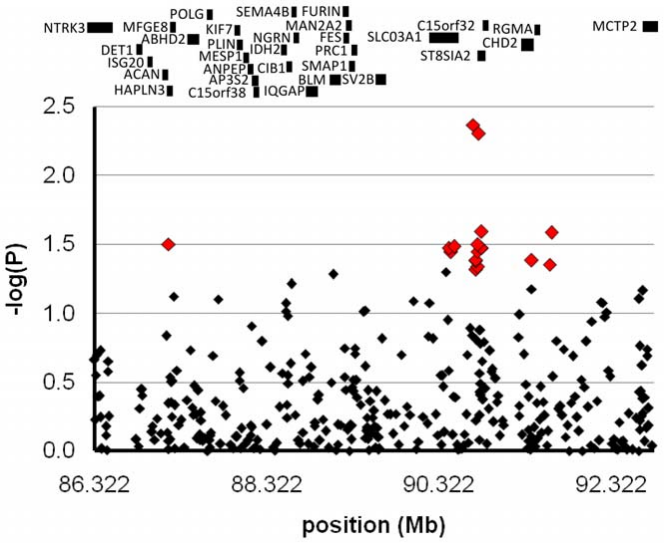
Association analysis and gene map of 6.5 Mb candidate locus. −Log of the *P* values of genotyped SNPs are represented on the y-axis with chromosome position in kilobases on the x-axis. The red diamonds indicate the *P* values less than 0.05. The relative positions of protein coding genes in the locus are shown above.

The 349 successfully genotyped SNPs were tested for association with bipolar disorder (Figure 1). Nominally significant association (*P*<0.05) was found with 18 SNPs, in three main clusters across three adjacent genes *SLCO3A1*, *ST8SIA2*, and *C15orf32* (Table 1). Association with 14 of these SNPs held true when empirical *P* values were obtained through 10,000 replicate permutations (Table 1). The strongest single point association signals were found with SNP rs4586379 (*P* = 0.0043, empirical *P* = 0.006), which lies 16 kb upstream from *ST8SIA2*, and rs2168351 (*P* = 0.0045, empirical *P* = 0.007) in the fourth intron of *ST8SIA2*.

**Table 1:** SNPs with nominally significant *P* values (<0.05) and their empirical *P* values.

SNP	location in gene	bp position (hg18)	P-value	Empirical P-value	SNP^b^	MAF (cases)	MAF(controls)	odds ratio (95% CI)	Gentrain Score	Cluster Separation	% genotypes called
rs2280468	AGC1 intron 2	87182560	0.031	0.030^a^	A/G	0.248	0.319	0.70 (0.51–0.96)	0.71	0.47	100
rs871167	SLCO3A1 intron 2	90435137	0.034	0.046^a^	A/G	0.401	0.325	1.39 (1.06–1.93)	0.74	0.65	100
rs7175812	SLCO3A1 intron 4	90464205	0.036	0.035^a^	G/A	0.192	0.134	1.53 (1.07–2.38)	0.92	0.92	100
rs6496898	SLCO3A1 intron 9	90502207	0.033	0.035^a^	G/A	0.315	0.244	1.43 (1.04–1.99)	0.90	0.72	100
rs4586379	16 kb upstream of ST8SIA2	90722031	0.004	0.006^a^	G/A	0.190	0.278	0.61 (0.43–0.85)	0.76	0.35	100
rs2035645	ST8SIA2 intron 1	90747330	0.042	0.060	A/C	0.475	0.400	1.36 (1.00–1.78)	0.70	0.59	100
rs4777974	ST8SIA2 intron 1	90753294	0.041	0.050	G/A	0.465	0.391	1.36 (1.00–1.79)	0.80	0.80	100
rs11637898	ST8SIA2 intron 1	90753854	0.048	0.064	A/G	0.449	0.522	0.75 (0.56–0.99)	0.75	0.63	100
rs11074070	ST8SIA2 intron 1	90767830	0.036	0.040^a^	C/A	0.435	0.513	0.73 (0.55–0.98)	0.87	0.95	100
rs3858917	ST8SIA2 intron 1	90772815	0.046	0.055	C/A	0.442	0.516	0.74 (0.56–0.99)	0.74	0.69	100
rs3784735	ST8SIA2 intron 2	90776333	0.032	0.034^a^	C/A	0.449	0.528	0.73 (0.54–0.97)	0.87	1.00	100
rs2168351	ST8SIA2 intron 4	90784726	0.005	0.007^a^	G/A	0.331	0.431	0.65 (0.49–0.89)	0.74	0.61	100
rs1455773	C15orf32 exon 1 (missense)	90816431	0.026	0.031^a^	A/G	0.340	0.264	1.44 (0.99–1.86)	0.69	0.31	99.7
rs13380006	C15orf32 intron 1	90817132	0.034	0.042^a^	A/G	0.338	0.266	1.41 (0.97–1.82)	0.79	0.82	100
rs12441404	C15orf32 intron 2	90817916	0.034	0.042^a^	A/G	0.338	0.266	1.41 (0.97–1.82)	0.76	0.56	100
rs4777760	RGMA intron 2	91403321	0.042	0.045^a^	A/G	0.438	0.513	0.74 (0.58–1.03)	0.80	0.63	100
rs7169541	No known gene	91612214	0.045	0.045^a^	G/C	0.368	0.441	0.74 (0.55–0.99)	0.67	0.42	100
rs7174649	No known gene	91634219	0.026	0.035^a^	A/G	0.120	0.178	0.63 (0.42–0.94)	0.89	0.98	100

a Significant pointwise *P* values derived empirically through 10,000 permutations (EMP1 *P*<0.05). Empirical *P* values for all SNPs are non-significant after multiple testing correction (EMP2 *P*>0.05).

b minor allele of each SNP is listed first.

Haplotypes were tested for association with bipolar disorder using a 3 SNP sliding window analysis, and global significant *P* values were found for a haplotype upstream of *ST8SIA2* (rs11634097, rs8027941 and rs4586379: omnibus *P* = 0.04, empirical *P* = 0.050) and for a haplotype with one SNP upstream of *ST8SIA2* and two within the first intron of the gene (rs4586379, rs2035645 and rs4777973: omnibus *P* = 0.026, empirical *P* = 0.056). Significant global association was also found for a 3 SNP haplotype across intron 2 of the *SLCO3A1* gene (rs1400786, rs995002 and rs8031518; omnibus *P* = 0.034, empirical *P* = 0.031), upstream of *ST8SIA2*.

Implementing regression-based conditional haplotype association testing, we investigated various haplotypes with the individually associated *ST8SIA2* SNPs. Global significant association across a 6 SNP haplotype spanning 54 kb, starting 16 kb upstream of *ST8SIA2* through to the middle of intron 2, was found with SNPs rs4586379, rs2035645, rs4777974, rs11637898, rs11074070 and rs3784735 (*P* = 0.017). Three specific haplotypes had a minimum haplotype frequency greater than 10 percent, two of which demonstrated significant association with bipolar disorder: the most common haplotype AAGGAA (χ^2^ = 6.90, *P* = 0.0086, OR 1.47), had a higher frequency in cases (0.41) than in controls (0.31) constituting a putative bipolar risk haplotype; and its flip variant GCAACC (χ^2^ = 4.95, *P* = 0.026, OR 0.61), representing the third most common haplotype had a higher frequency in controls (0.19) than in cases (0.13), constituting a putative protective haplotype (Table 2).

**Table 2:** Summary of haplotype frequencies and associations for Australian and NIMH case-control cohorts and SMRI postmortem brain cohort.

cohort	haplotype	Freq (all)	Freq (cases)	Freq (controls)	χ^2^	P value	Odds ratio (95%CI)
Aus BP	risk^a^	0.351	0.405	0.313	6.46	**0.011**	1.473 (1.09–1.99)
Aus SZ	risk^a^	0.348	0.415	0.297	8.41	**0.004**	1.82 (1.2–2.74)
NIMH BP	risk^b^	0.399	0.432	0.398	5.91	**0.015**	1.29 (1.03–1.33)
NIMH SZ	risk^b^	0.395	0.421	0.396	2.30	0.129	1.09 (0.97–1.23)
SMRI (BP+SZ)	risk^a^	0.319	0.377	0.237	4.78	**0.029**	2.185 (1.05–4.56)
SMRI (BPonly)	risk^a^	0.319	0.357	0.237	2.24	0.134	1.865 (0.81–4.30)
SMRI (SZonly)	risk^a^	0.319	0.395	0.237	5.08	**0.024**	2.456 (1.08–5.58)
Aus BP	protective^c^	0.149	0.129	0.192	5.97	**0.015**	0.5857 (0.38–0.90)
Aus SZ	protective^c^	0.138	0.105	0.177	5.30	**0.021**	0.515 (0.29–0.92)
NIMH BP	protective^d^	0.169	0.166	0.185	2.83	0.090	0.87 (0.73–1.02)
NIMH SZ	protective^d^	0.167	0.169	0.176	0.68	0.408	0.938 (0.81–1.09)
SMRI (BP+SZ)	protective^c^	0.106	0.090	0.135	0.88	0.350	0.614 (0.22–1.67)
SMRI (BPonly)	protective^c^	0.106	0.067	0.135	2.03	0.155	0.366 (0.09–1.51)
SMRI (SZonly)	protective^c^	0.106	0.112	0.135	0.16	0.685	0.794 (0.26–2.44)

a haplotype defined by AAGGAA at rs4586379, rs2035645, rs4777974, rs11637898, rs11074070, and rs3784735.

b haplotype defined by TTAGT at rs4586379, rs8025225, rs11074066, rs11074067, and rs2035645.

c haplotype defined by GCAACC at rs4586379, rs2035645, rs4777974, rs11637898, rs11074070, and rs3784735.

d haplotype defined by CCCCG at rs4586379, rs8025225, rs11074066, rs11074067, and rs2035645.

Statistical methods to account for type I (false positive) error rates after multiple comparisons and are generally conservative, and may also increase the chance of type II (false negative) errors, particularly for small effect sizes in large numbers of SNPs [15]. To correct for multiple comparisons, we determined the effective number of independent tests using the simpleM method [16], and LD based pruning in PLINK [17], followed by Šidák correction. The simpleM method yielded 295 independent tests, and recursive SNP removal in PLINK yielded 174–210 independent SNPs using the recommended variance inflation factor values (VIF range = 1.5–2.0). No SNPs or haplotypes passed the Šidák correction threshold to exclude type I error after multiple testing correction (at α = 0.05, simpleM: *P*<0.00017; PLINK: *P*<0.00028). However, as replication is the ‘gold standard’ to refute type I error, we sought to determine whether the observed association in the LD block containing *ST8SIA2* was replicable in other bipolar disorder cohorts, and to determine whether this same LD risk haplotype in *ST8SIA2* may also increase risk in other cohorts that included schizophrenia patients.

### Testing for association in an Australian schizophrenia cohort

The 6 SNPs comprising the risk haplotype were genotyped in an Australian schizophrenia cohort, and haplotype phasing and association analysis conducted in PLINK. We found that the specific AAGGAA risk haplotype was significantly over-represented in schizophrenia cases compared to controls when a conditional analysis was performed (χ^2^ = 8.04, *P* = 0.004, OR = 2.62) (Table 2). Similarly, the protective haplotype was over-represented in controls on conditional haplotype analysis (χ^2^ = 7.25, *P* = 0.007, OR 0.768). Single SNP analysis did not yield significant evidence for association (p values all ≥0.075).

### Replication in the NIMH bipolar disorder and schizophrenia cohorts

**Figure 2:**
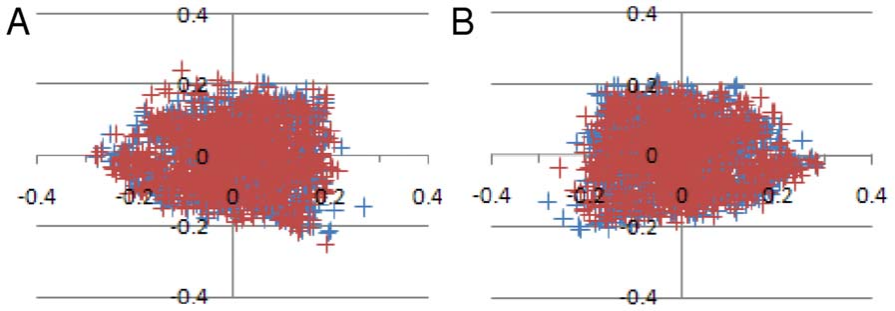
Multidimensional scaling of GAIN data showing no evidence of population stratification. A) bipolar disorder case control cohort; B) schizophrenia case control cohort. Cases and controls are represented by the red and blue crosses respectively.

Using GWAS data downloaded from GAIN, we determined whether the over representation of the identified risk haplotype was replicable. First, we examined population stratification using whole genome SNP data via PLINK, and only one significant cluster was identified for both the bipolar and schizophrenia cohorts (Figure 2). Permutation tests for between group differences of identity by state (IBS) showed that cases in either cohort did not show more IBS on average than expected by chance (bipolar: p = 0.208; schizophrenia: p = 0.552). Next, we examined whether the Australian risk haplotype was assessable using SNP genotypes from the Affymetrix 6.0 chip. Of the six SNPs making up the Australian risk haplotype, only two were genotyped in the NIMH data: SNP_A-8471284 (rs4586379 at 90722031 bp, with T representing the risk allele and C the protective allele), and SNP_A-2278085 (rs2035645 at 90747330 bp, with T representing the risk allele and G the protective allele). Accurate representation of the specific 6 SNP risk haplotype in the NIMH data was not possible by imputation, as rs11074070 was not genotyped in HAPMAP3 reference panel (release 28) and rs11637898 performed poorly, with 23.5% of the sample failing to be imputed accurately.

**Figure 3:**
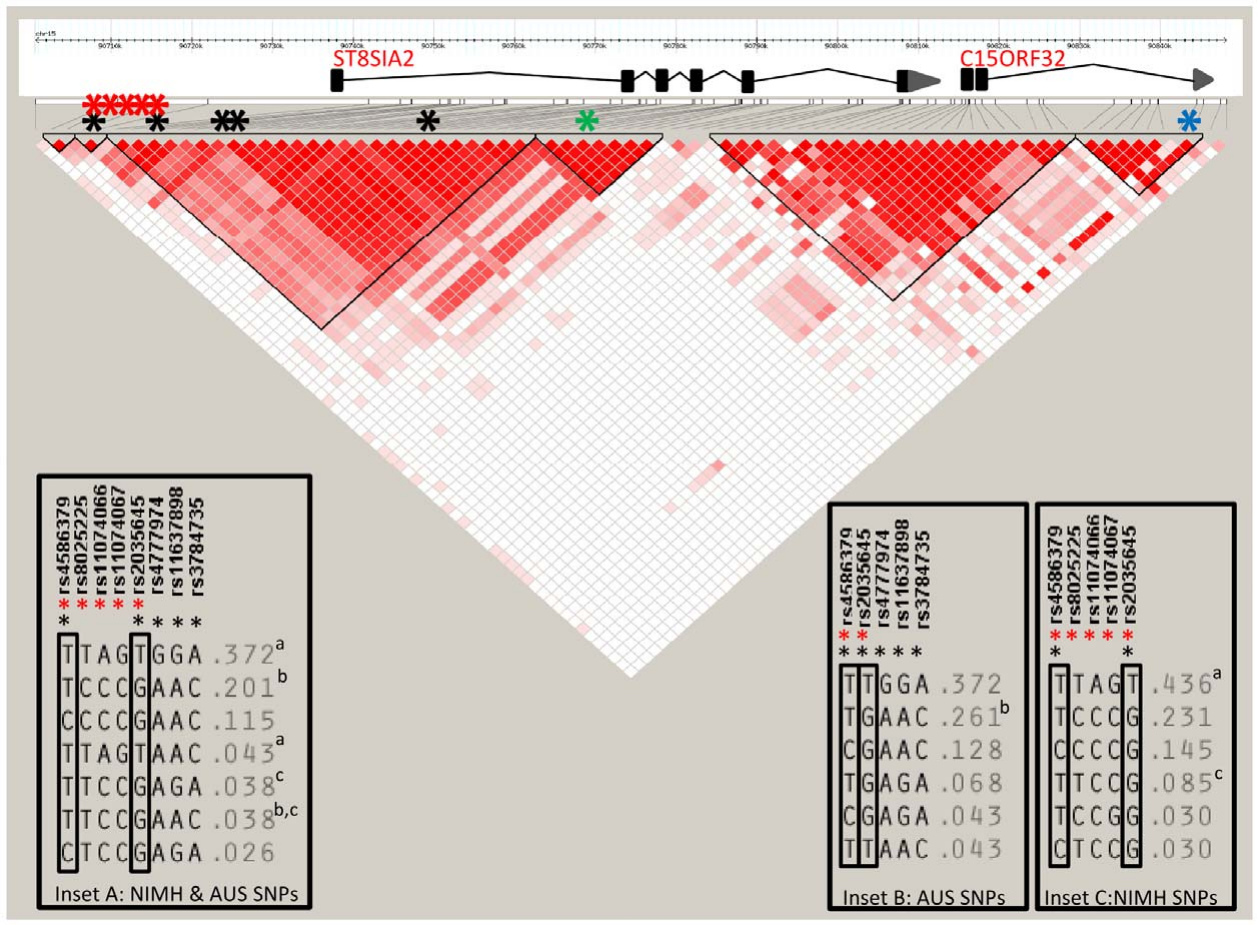
Linkage disequilibrium structure of the ST8SIA2 and C15ORF32 region, spanning 132 kb, in Caucasian Europeans from the CEPH collection. LD blocks are indicated in black triangles, with red shading indicating high LD (D′) for CEU genotypes downloaded from HAPMAP3 (release #28; 90720–90845 kb, NCBI build 36.1). Locations of SNPs which make up the risk haplotype in the Australian bipolar disorder cohort are indicated by the black asterisks. Affymetrix SNPs used to replicate the haplotype association in the NIMH bipolar and schizophrenia case-control samples are indicated by the red asterisks. Insets A–C show the relationships between common haplotypes (frequency greater than 2%) in the CEPH genotype data using different SNP combinations. Haplotypes indicated by the superscript letters in inset A (when both NIMH and Australian SNPs are considered simultaneously) indicate those haplotypes which are indistinguishable when using only the Australian SNPs (inset B), or the surrogate NIMH SNPs (inset C). The strongly associated SNPs from genome wide association studies of bipolar disorder in the Han Chinese (rs8040009) [26], and of autism in Caucasians (rs3784730) [14] are indicated by the blue and green asterisks respectively.

To determine whether a different combination of SNPs could be used to define a surrogate “risk” haplotype in the NIMH affymetrix data, we investigated the haplotype structure of the associated LD block in HAPMAP3 data from Caucasian Europeans (release 28), where both the Australian and NIMH SNPs had been genotyped (Figure 3). With the addition of three intermediate Affymetrix SNPs (SNP_A-1920907 (rs8025225) at 90741904 bp, SNP_A-1787148 (rs11074066) at 90745868 bp and SNP_A-4204154 (rs11074067) at 90747302 bp) we were able to observe three common haplotypes (MAF>0.1), each of which had a similar frequency distribution in individuals genotyped with both sets of markers (Figure 3). Given the similarities in haplotype frequencies, and the informativity of rs4586379 and rs2035645 in defining the risk and protective haplotypes, the 5 SNP Affymetrix haplotype was considered to be a reasonable surrogate for the Australian 6 SNP haplotype.

Using the five Affymetrix SNPs to phase haplotypes (average posterior probability of haplotype phases given genotype data = 0.961±0.085), we found that the frequencies of the three main haplotypes were comparable between the NIMH sample and those observed with 6 SNPs in the Australian population (0.399 vs 0.351, 0.237 vs 0.228 and 0.169 vs 0.149). The most common haplotype (TTAGT, equivalent to the risk haplotype identified in the Australian cohort) was significantly over represented in the NIMH bipolar disorder cases compared to controls (frequency = 0.432 vs 0.398, χ^2^ = 5.91, df = 1, *P* = 0.0151, OR = 1.29), using a conditional haplotype test (Table 2). This haplotype also showed the same direction of effect in the NIMH cases with schizophrenia compared to controls (frequency = 0.421 vs 0.396), although it did not reach statistical significance (χ^2^ = 2.3, df = 1, *P* = 0.129, OR = 1.09).

### *ST8SIA2* developmental profile

**Figure 4:**
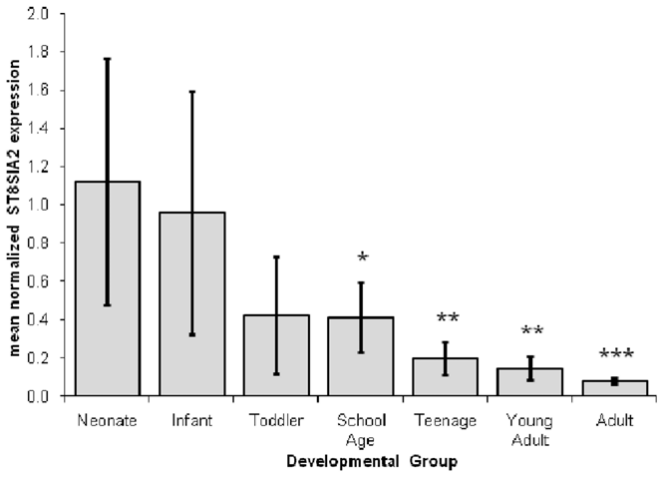
The mean normalized quantity for each developmental group. The age range in years and number of subjects per group (n) were: neonate 0.11–0.24 (n = 10); infant 0.32–0.91 (n = 13); toddler 1.58–4.86 (n = 9); school age 6.88–12.97 (n = 8); teenage 15–17.82 (n = 8); young adult 20.14–25.38 (n = 8); adult 35.99–48.69 (n = 7). Error bars indicate one standard deviation from the mean. Significant group differences from post-hoc Fisher LSD tests comparing neonates to the six other developmental age groups are indicated, where one asterisk represents *P*<0.001, two asterisks represent *P*<0.0001 and three asterisks represent *P*<0.00001.

Analysis of microarray data [18] showed a significant negative correlation between age and *ST8SIA2* gene expression (r = −0.93, *P*<0.00001), with a steady decrease from birth through to adulthood. This was confirmed by RT-PCR, with high levels of expression seen in the neonate and infant groups, and a gradual decrease in expression from toddler to adult (Figure 4). Importantly, we found that despite being developmentally regulated, *ST8SIA2* mRNA is detectable in the adult DLPFC.

### *ST8SIA2* expression in adult bipolar disorder and schizophrenia patients

Consistent with the Australian and US based case-control cohorts, there was a higher frequency of risk haplotypes in SMRI cases with bipolar and schizophrenia compared to controls (36.7%, 38.9% and 22.8% respectively) and lower frequency of protective haplotypes (8.8%, 11.1% and 14.2% respectively) (Table 2).

As expected, there was a significant negative correlation of *ST8SIA2* expression with patient age (r = −0.392, *P* = 5.5×10^−5^) and duration of illness (r = −0.516, *P* = 9.3×10^−6^), which are highly correlated measures (r = 0.664, *P* = 4.8×10^−6^). As a result, age was included in a linear regression against *ST8SIA2* expression, and the residuals used in further analysis. There was no significant correlation of *ST8SIA2* mRNA with other demographic variables tested (including post-mortem interval, brain pH, refrigerator interval, duration of illness, age of onset or lifetime antipsychotic use), nor were there any significant effects (by t-test) of sex, brain hemisphere, smoking, or psychotic features.

**Figure 5:**
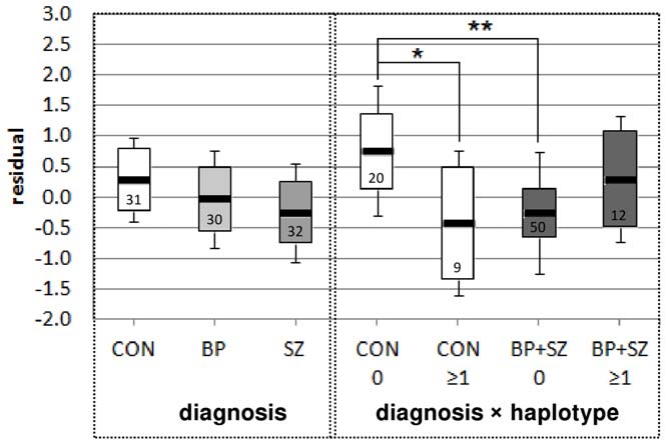
The effect of protective haplotype on *ST8SIA2* gene expression in adult post-mortem DLPFC. Normalised and transformed *ST8SIA2* expression was regressed against age, and standardized residuals were used to compare the effect of haplotype in Caucasian brains from the Stanley Medical Research Institute Array cohort. The mean values for each group are represented by horizontal black bars, and boxes represent the 95% confidence intervals of each group (± standard errors), with the numbers of individuals in each group indicated within the boxes. One individual homozygous for the protective haplotype was identified, and was included in the ≥1 copy group. There was no significant overall effect of diagnosis on expression (three-way ANOVA: F(2, 94) = 0.486, p = 0.617), but a trend towards lower *ST8SIA2* expression in schizophrenia cases compared to controls (ANOVA: F(1, 61) = 2.737, p = 0.103; combined diagnostic group ANOVA: F(1, 91) = 1.948, p = 0.166). Significant diagnosis×haplotype effects were observed for the protective haplotype in control versus the combined diagnostic group (ANOVA: F(1, 87) = 6.031, p = 0.016). Post-hoc comparisons showed significant reductions in *ST8SIA2* expression in non-haplotype carriers in cases compared to non-haplotype carrier controls (Fisher LSD: combined diagnoses p = 0.007, indicated by double asterisk), as well as control carriers versus control non-carriers (Fisher LSD: p = 0.034, indicated by single asterisk).

There was no main effect of diagnosis on *ST8SIA2* expression (three-way ANOVA: F(2, 94) = 0.486, p = 0.617), but a trend towards lower *ST8SIA2* expression in schizophrenia cases (ANOVA: F(1, 61) = 2.737, p = 0.103) (Figure 5A). There was no significant diagnosis×haplotype effect for the risk haplotype (*P* values all>0.744). However a significant diagnosis×haplotype effect was observed with the protective haplotype (ANOVA: bipolar versus control: F(1,55) = 6.462, *P* = 0.014; combined diagnostic group: F(1,87) = 6.031, *P* = 0.016) (Figure 5), with suggestive effects in the control versus schizophrenia comparison (F(1,57) = 3.825, *P* = 0.055). Post-hoc comparisons showed significant reductions in *ST8SIA2* expression in case non-carriers of the protective haplotype compared to control non-carriers (Fisher LSD: combined diagnoses p = 0.007 (Figure 5); bipolar disorder or schizophrenia only p = 0.023 or 0.006 respectively), as well as control carriers versus control non-carriers (Fisher LSD: p = 0.034). Most of these effects remained significant or suggestive after Bonferroni correction for multiple comparisons (corrected post-hoc LSD p values = 0.041, 0.14, 0.036, 0.21 respectively).

## Discussion

Multiple studies have shown linkage of psychiatric illness to chromosome 15q25-26, including bipolar disorder [12], bipolar disorder with psychosis [19], bipolar disorder and schizophrenia [20], and recurrent early onset depression [21], [22], indicating that this locus may harbour a generalized susceptibility gene for both psychotic and mood disorders. Through a fine mapping study of a bipolar disorder linkage peak [12], we identified a specific risk haplotype within *ST8SIA2* which was over-represented in cases in three independent cohorts: 1) the Australian bipolar disorder case-control cohort; 2) the Australian schizophrenia case-control cohort; and 3) the American NIMH bipolar disorder case-control cohort. While the results from a fourth cohort – the American NIMH schizophrenia cohort – were not significant, the same direction of effect was observed. Taken together, these findings indicate that *ST8SIA2*, a facilitator of neural plasticity which is highly expressed early in human brain development, may play a role in the genetic susceptibility for both bipolar disorder and schizophrenia.

Variants in the promoter of *ST8SIA2* have previously shown association with schizophrenia in Japanese and Chinese cohorts [13], [23], after the gene was selected as a functional candidate due to its interaction with *NCAM*, which has also previously shown association with bipolar disorder [24]. Indeed our study has also identified genetic variants in the promoter region that are associated with bipolar disorder, albeit with different alleles. The low frequency of the associated alleles from the Asian studies in the Caucasian population may explain the failure to replicate association with those specific promoter variants in an Italian cohort [25] and our Australian cohort (data not shown). Moreover, a GWAS of bipolar disorder in Han Chinese found strong association (*P*<6×10^−6^) with two SNPs which lie 30 kb downstream of *ST8SIA2* in the *C15orf32* gene [26]. Long range linkage disequilibrium between the two *C15orf32* SNPs with markers towards the 3′ end of *ST8SIA2* (*D′*≥0.916) (Figure 2) suggests these independent signals may converge functionally on a single genetic effect.

An association study of 15q25-26 for recurrent early onset depression showed nominal association with *NTRK3* and the genes flanking *ST8SIA2: SLCO3A1* and *C15orf32*; but not *ST8SIA2* itself [21], [22]. Interestingly, a recent GWAS for autism spectrum disorder using 1314 families with the verbal subtype identified a significant signal (P = 5.37×10^−8^) for rs3784730 in intron 4 of *ST8SIA2* [14], which lies 3.4 kb from our associated single SNP marker rs2168351. As autism is thought to be part of a broader spectrum of neurodevelopmental disorders, and shares genetic and clinical overlaps with both schizophrenia and bipolar disorder [27], it is possible that *ST8SIA2* may be a generalised susceptibility gene for all three illnesses.

Functionally, *ST8SIA2* is an attractive candidate for mental illnesses with neurodevelopmental pathology. The sialyltransferases, *ST8SIA2* and *ST8SIA4*, are principally responsible for the post-translational glycosylation of the neuronal cell adhesion molecule (NCAM), which in its polysialylated form (PSA-NCAM), plays an important role in processes such as neuronal migration and axon pathfinding, synaptogenesis and plasticity [28], [29]. This occurs via masking the adhesive properties of NCAM through steric repulsion after the addition of large hydrophilic glycopolymers, which facilitates movement through intracellular space for both neurons and neuronal processes [30]. In addition, PSA-NCAM has been implicated in hormone response via the dopamine D2 receptor [31], [32], in regulation of circadian cues via photic entrainment of the suprachiasmatic nucleus [33], and in stress response and learning and memory [30]. The high expression of *ST8SIA2* early in brain development, when neuronal networks are first being established, and the persistence of PSA-NCAM in regions of neurogenesis in the adult brain [34], [35], suggests that aberrant temporal or spatial expression of *ST8SIA2* may disrupt the highly coordinated early development of brain connectivity and affect plasticity of established neuronal networks [29]. Some adults with schizophrenia have a 20–95% reduction of PSA-NCAM immunoreactive cells in the hippocampus that is not accounted for by changes in NCAM expression [36], suggesting a polysialylation defect which may continue into adulthood. Mouse knockouts of sialytransferase have abnormal fear responses [37] and misguidance of axonal fibre tracts in the anterior commissure, corpus callosum and internal capsule [29]. While we did not find an effect of the risk haplotype on global *ST8SIA2* mRNA in the adult DLPFC, the absence of the protective haplotype in cases appears to reduce global *ST8SIA2* expression compared to controls, which is conceptually consistent with reduced neuroplasticity. This result should be considered preliminary, and the opposing effects of the protective haplotype in controls may indicate that additional interacting factors with disease specific expression may be required to fully understand the mechanism through which the protective haplotype exerts its effects. It may be that alterations specific to the risk haplotype occur earlier in development, or affect particular splicing isoforms of *ST8SIA2*, neither of which were assessed in the current study. Further investigations of the effects of the risk and protective haplotypes on gene function are required, and are currently underway.

Finally, we acknowledge that there are limitations to our study. Firstly, while our discovery sample is enriched for individuals from families which may share a susceptibility gene on 15q25-26 due to inclusion of individual pedigree members from families showing linkage to this genomic region, our discovery sample is small in the current context of large scale genome-wide association studies. This is problematic for two reasons: 1) the power of our cohort to detect significant association which exceeds multiple testing correction penalties is limited; and 2) the results from our fine-mapping study may not be generalizable to other cohorts which are not enriched for the 15q25-26 susceptibility gene/s. We have attempted to reconcile these problems by replicating our findings in additional independent cohorts, with which we have had some success. While the results from the NIMH bipolar disorder cohort are significant, the effect size is smaller than in our discovery cohort. This is not surprising given the ascertainment of the samples, the difference in genotyped SNPs across the studies, and the difference in sample size. Our cohort is largely derived from extended families with a high density of illness, hence may contain rarer and more highly penetrant risk genes than the NIMH cohort, which is largely derived from singleton cases whose illness may be more sporadic in nature. In spite of this, we did observe an over-representation of a surrogate *ST8SIA2* risk haplotype in the NIMH data, suggesting that *ST8SIA2* may contribute to disease risk. However this result should be considered preliminary and requires further replication. Imputation or direct genotyping of the SNPs pertaining to the specific risk haplotype in the NIMH cohort would circumvent problems with using surrogate haplotypes, which are less than ideal. Secondly, we acknowledge that our primary fine-mapping findings do not withstand correction for multiple comparisons, and hence should be interpreted with caution. Thirdly, as we do not have genome-wide SNP data for the Australian samples, we are unable to directly test for population stratification, nor determine how this may affect our findings. While we have ensured our sample is racially homogeneous through demographic information, it is possible that the association in our discovery sample is affected by population sub-structure. Nonetheless, we were able to confirm that population stratification did not affect the association signal in the NIMH GWAS data.

We propose that *ST8SIA2* is a generalised susceptibility gene for mental illnesses with neurodevelopmental origins. The role of sialyltransferase as a facilitator of neuronal migration and synaptogenesis makes this gene an appealing functional candidate for bipolar disorder, schizophrenia and autism, each being complex genetic syndromes with overlapping constellations of clinical symptoms. The involvement of *ST8SIA2* fits with a polyfactorial model of susceptibility for mental illness, where defects in the development of brain connectivity early in life, caused by inheritance of risk variants in *ST8SIA2* which reduce glycosylation of NCAM resulting in axonal misguidance and reduced neuronal plasticity, may render the brain more susceptible to secondary genetic and environmental insults later in life.

## Materials and Methods

### Ethics statement

This study was carried out in accordance with the latest version of the Declaration of Helsinki after specific approval by the University of New South Wales Human Research Ethics Committee (Australian bipolar disorder cohort: HREC # 04144 and 10078), and the Wolston Park Hospital Institutional Ethics Committee (Australian Schizophrenia cohort). Developing human post-mortem DLPFC tissue was obtained from the University of Maryland Brain Tissue Bank for Developmental Disorders (NICHHD contract # NO1-HD8-3283). Samples from the Stanley Medical Research Institute were collected and distributed under approval for the Department of Psychiatry, Uniformed Services University of the Health Sciences, Bethesda USA. Genotype data were obtained with approval from the National Institutes of Health Data Access Committee, after approval by the Johns Hopkins University School of Medicine institutional review board.

### Australian Bipolar disorder cohort

The Australian bipolar disorder case-control cohort was mostly selected from families previously used for linkage analysis and was recruited through the Mood Disorders Unit and Black Dog Institute, Prince of Wales Hospital/School of Psychiatry, University of New South Wales, Australia. Families were ascertained through a bipolar I disorder proband using the Family Interview for Genetic Studies (FIGS), where a relative with schizophrenia in the absence of mood disturbance resulted in family exclusion. One affected individual, plus a spouse or completely unrelated pedigree member, was selected from each family for cases and controls respectively. Gender was matched across the groups, although the control group was older to reduce the likelihood of a later diagnosis. The rest of the cases were recruited from a specialized bipolar disorder clinic [38]. In total, 50.5% of the sample had psychotic features, 70% had a family history of bipolar disorder, and 87% had a family history of bipolar disorder or depression. All individuals gave written informed consent, and were assessed using the Diagnostic Interview for Genetic Studies (DIGS) by experienced medical practitioners, psychologists and psychiatric nurses trained in this instrument [39]. Information obtained from FIGS, DIGS and medical records was used to generate best-estimate Research Diagnostic Criteria (RDC) diagnoses for Bipolar I disorder (BPI:n = 158), Bipolar II disorder (BPII:n = 41), Schizoaffective Disorder-Manic type (SZ/MA:n = 10), Major Depressive Disorder (MDD:n = 10) or unaffected controls (n = 166). All genotyped individuals were Caucasian and almost entirely of Anglo-Celtic descent and 50% were female. Demographic information for the cohort is presented in Table 3. The study was approved by the Human Research Ethics Committee of the University of New South Wales.

**Table 3:** Demographic information for bipolar disorder case control cohort.

	male	female	total	% male
**total controls**	**89**	**77**	**166**	**0.54**
average age of controls (years±SD)	61.2±13.0	58.9±14.0	60.3±13.5	
Bipolar I	68	90	158	0.43
Bipolar II	28	13	41	0.68
Schizoaffective disorder-manic type	7	3	10	0.70
Recurrent Unipolar depression	2	8	10	0.20
**total cases**	**105**	**114**	**219**	**0.48**
average age of cases (years±SD)	47.3±15.1	46.7±15.3	46.7±15.3	
age of onset for depression (years±SD)			23.9±10.9	
age of onset for mania (years±SD)			28.1±11.3	
number of cases with psychotic features			92	
number of cases with family history (bipolar, depression, either)			119, 94, 151	

### Australian Schizophrenia cohort

Samples were selected from a larger cohort (n = 310), initially recruited for the Australian national prevalence study of psychosis [40], for which diagnoses were assigned using the Diagnostic Interview for Psychosis [41]. Individuals were included in the current study if they were Caucasian, had a confirmed diagnosis of schizophrenia and a DNA sample available. In total, 128 cases (85 men and 43 women) with a DSM-III-R [42] diagnosis of schizophrenia and 128 age-, gender- and ethnically-matched controls were included.

### NIMH bipolar disorder and schizophrenia cohorts

Genome wide association study data was downloaded from the Genetic Association Information Network (GAIN) through the US based National Institutes of Health. Filtered genotypes generated on the Affymetrix 6.0 SNP from European ancestry samples were used from: 1) the GWAS of Bipolar Disorder version 3 study, which contained 1034 controls and 1021 patients with bipolar disorder and related disorders, including any psychiatric disorder as defined in DSM-IV or ICD-10; and 2) the GWAS of Schizophrenia version 2 data, which contained 1378 controls and 1172 cases with schizophrenia and related conditions, including schizoaffective disorder, acute psychoses, bipolar disorder, major depressive disorder, or “Cluster A” personality disorders (schizotypal, schizoid, paranoid).

### SNP selection and genotyping of the linkage interval

The 6.2 Mb linkage interval defined by microsatellite markers D15S979 and D15S816 included only the 5′ end of the *NTRK3* gene, so the screened region was expanded to 6.5 Mb (86.32–92.82 Mb) to cover the *NTRK3* gene. The 6.5 Mb region contained ∼22,000 known SNPs, (7,315 in HAPMAP release 28, Caucasian Europeans), from which 376 were selected using the Illumina GoldenGate Genotyping Assay on the BeadArray platform (Illumina Inc., San Diego USA). Selected SNPs had a score of <0.6 in the score file supplied by Illumina, and a minor allele frequency ≥0.1. They either tagged common (>5% frequency in Caucasians) major haplotype blocks around known genes, as determined through HAPLOVIEW; covered other putative functional regions with high sequence conservation (28-way cross-species alignment; UCSC Genome Bioinformatics); or covered major gaps between other selected SNPs, such that the average inter-SNP distance was 15 kb across the 2.5 Mb high priority region, or 20 kb across the rest of the 6.5 Mb region. Six extra putative functional SNPs within *ST8SIA2* were selected, which were predicted bioinformatically [43] to alter splice donor/acceptor sites. While the SNPs selected for genotyping represent only 5% of the known SNP variation in the region (HAPMAP release 28, Caucasian Europeans), through the underlying linkage disequilibrium structure they tag 53.6% of all common variation in the interval (MAF>0.1 at r^2^>0.80), and tag 66.2% of common variation within genes (mean max r^2^ = 0.94).

Scan data from the BeadArray was interpreted using the BeadStudio-GT module (Illumina Inc, San Diego USA), where SNP allele calls were clustered and assessed for genotype quality, and genotype reports generated. SNPs and samples that did not pass standard quality control measures, including clustering distribution and missingness (per marker or per sample) were removed from further analyses.

### Association and haplotype analysis

Statistical analyses were performed with PLINK v1.02 software [17]. SNPs failing the Hardy-Weinberg exact test at a 0.001 significance threshold were removed. Individuals with high SNP genotype failure rates (>10%) were excluded. Association analysis for bipolar disorder was performed using a broad disease model where individuals diagnosed with BPI, SZMA, BPII or MDD were all classified as affected (n = 218). To devise empirical *P* values for SNP and haplotype association, ten thousand permutations for each SNP were performed using the *–mperm* option.

Multimarker haplotype analysis was conducted using probabilistically inferred haplotypes for each individual, generated using a standard E-M algorithm in PLINK [17]. For each imputed haplotype, the average posterior probability of haplotype phases given genotype data was generated, with average values of 0.95±0.10 for the Australian bipolar disorder case-control cohort, 0.93±0.11 for the Australian schizophrenia case-control cohort, 0.94±0.12 for the Stanley Medical Research Institute post-mortem brain cohort, 0.96±0.09 for the NIMH bipolar disorder cohort and 0.96±0.09 for the NIMH schizophrenia cohort. Haplotype association analysis was performed using the –*hap-assoc* option with a sliding windows (*–hap-window* 3) or specified haplotype (*–hap-snps*) approach with standard association testing. Conditional regression-based association using the –*chap* option was performed on specific haplotypes of interest.

### *ST8SIA2* expression profiling over normal brain development

Because *ST8SIA2* is expressed early in development [44], we determined the temporal expression profile of *ST8SIA2* using a cohort of 63 normal human brains at different stages of development, consisting of 10 neonates (aged between 0.11–0.24 years); 13 infants (0.32–0.91 years); 9 toddlers (1.58–4.86 years); 8 school age children (6.88–12.97 years); 8 teenagers (15–17.82 years); 8 young adults (20.14–25.38); and 7 adults (35.99–48.69 years). A previous genome-wide transcription profiling microarray experiment of the dorsolateral prefrontal cortex (DLPFC) from this cohort [18] included a probe specific for *ST8SIA2* exon 6 (239537_at), which showed a marked reduction in expression over brain development. We validated the microarray findings by quantitative real-time PCR after reverse transcribing RNA using the SuperScript III First-Strand Synthesis kit with random hexamers (Invitrogen). Relative *ST8SIA2* transcript levels were determined using a TaqMan probe spanning exons 5–6 (Hs00544029_m1) on the ABI 7900HT PCR by the relative standard curve method using standard quantitative techniques [45]. *ST8SIA2* expression was then normalized to the geometric mean of two housekeeping genes, *HMBS* (Hs00609297_m1) and *GUSB* (Hs99999908_m1), neither of which changes over development. Statistical analysis was done by two-way ANOVA with age as an independent factor, and post hoc Fisher LSD.

### *ST8SIA2* expression in bipolar disorder and schizophrenia patients with the associated genetic risk variants

To determine whether the *ST8SIA2* risk or protective haplotypes have an effect on gene expression in brains of adult patients with bipolar disorder or schizophrenia, we used DNA and RNA extracted from 102 brains of Caucasian patients with bipolar disorder (n = 33), schizophrenia (n = 34) and controls (n = 35) from the Stanley Medical Research Institute (SMRI) Array cohort. The 6 SNPs comprising the risk haplotype were genotyped manually via restriction digest (rs4586379, rs2035645, rs11074070, rs3784735 with *XbaI*, *BstEII*, *HpyCH4IV* and *AlwNI* respectively) or direct sequencing (rs4777974, rs11637898), and haplotypes inferred using PLINK [17]. *ST8SIA2* gene expression was determined from DLFPC cDNA, using the methods described above and normalizing to the geometric mean of three housekeeper RNAs. Individual outliers were identified if they were greater than two standard deviations above or below their diagnostic group means, resulting in the exclusion of 5 samples from further analysis (3 controls, 2 schizophrenia cases). The effect of demographic variables, diagnosis and genotype on *ST8SIA2* gene expression was determined by correlation, linear regression and factorial ANOVA. The distribution of *ST8SIA2* expression was positively skewed, and was square root transformed to normalize the distribution to allow application of parametric statistics.
